# MicroRNAs as Regulator of Signaling Networks in Metastatic Colon Cancer

**DOI:** 10.1155/2015/823620

**Published:** 2015-05-06

**Authors:** Jian Wang, Yong Du, Xiaoming Liu, William C. Cho, Yinxue Yang

**Affiliations:** ^1^Human Stem Cell Institute of the General Hospital, Ningxia Medical University, Yinchuan 750004, China; ^2^Department of Colorectal Surgery, The General Hospital of Ningxia Medical University, Yinchuan 750004, China; ^3^Department of Clinical Oncology, Queen Elizabeth Hospital, Kowloon, Hong Kong

## Abstract

MicroRNAs (miRNAs) are a class of small, noncoding RNA molecules capable of regulating gene expression translationally and/or transcriptionally. A large number of evidence have demonstrated that miRNAs have a functional role in both physiological and pathological processes by regulating the expression of their target genes. Recently, the functionalities of miRNAs in the initiation, progression, angiogenesis, metastasis, and chemoresistance of tumors have gained increasing attentions. Particularly, the alteration of miRNA profiles has been correlated with the transformation and metastasis of various cancers, including colon cancer. This paper reports the latest findings on miRNAs involved in different signaling networks leading to colon cancer metastasis, mainly focusing on miRNA profiling and their roles in PTEN/PI3K, EGFR, TGF*β*, and p53 signaling pathways of metastatic colon cancer. The potential of miRNAs used as biomarkers in the diagnosis, prognosis, and therapeutic targets in colon cancer is also discussed.

## 1. Introduction

Colon cancer is one of the most commonly diagnosed cancers and a leading cause of cancer death worldwide. About 1.2 million new cases are diagnosed and it causes 0.6 million deaths annually around the world, of which cancer metastasis is the main cause of death [1]. It has been revealed that approximately one-third of patients with colon cancer have synchronous or metachronous metastasis. The five-year overall survival rate of patients with primary colon cancer can be up to 80–90%, despite it being reduced to 40–60% in patients with advanced nonmetastatic tumors. It is further decreased to 5–10% in patients with metastatic tumor [2]. As the survival rate of patients with colon cancer can be increased with effective treatment in its nonmetastatic phase, an extensive study of the underlying molecular networks of colon cancer metastasis is important for the discovery of novel prognostic molecular markers for colon cancer diagnosis and also for the development of effective targeted therapy for patients with metastatic colon cancer.

MicroRNAs (miRNAs) are a class of noncoding, small RNA molecules found in both prokaryotes and eukaryotes. The biogenesis of miRNA begins from the transcription of miRNA gene by RNA polymerase II to primary miRNA (pri-miRNA) containing hundreds of nucleotides length of RNA with a stem loop structure. The pri-miRNA can then be cleaved by Drosha from the nonloop end to form miRNA precursor (pre-miRNA). The pre-miRNA is a double-stranded hairpin structure of RNA with 60–70 bp in length [3], which can be further processed into matured miRNA by RNaseIII dicer, ultimately form RNA-induced silencing complex (RISC) after exported to cytoplasm by exportin-5 [4]. RISC can functionally inhibit gene expression by binding to the 3′ untranslated region (3′ UTR) in target mRNA, which is degraded if the miRNA:mRNA complex complementarity is perfect, or the translation is suppressed if the complementarity is not perfect. Recent studies have demonstrated that miRNA expression profiles are altered in tumors compared with adjacent normal tissues in a variety of cancers. The altered miRNA expression profile has been proposed to correlate with the stage and survival in patients with various cancers, including colon, lung, breast, and pancreatic cancers [5]. In this context, miRNAs can play a functionality of either tumor suppressors or oncogenes (oncomirs) [6]. Therefore, miRNAs may be utilized as novel targets for anticancer treatment.

**Figure 1:**
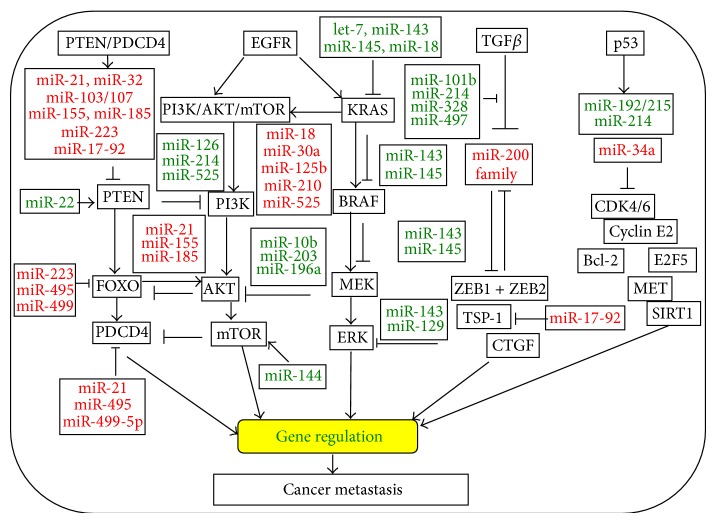
An illustration represents the overview of microRNAs (miRNAs) and their targets involving the key signaling pathways in metastatic colon cancer. The depicted miRNAs affect important factors of colon cancer development and malignancy, such as PTEN/PDCD4, EGFR/KRAS, EGFR/mTOR, TGF*β*, p53, and EMT transcription factors. miRNAs are able to regulate gene expressions and target the signaling pathways for controlling the proliferation, metastasis, chemoresistance, and survival of colon cancer cells. miRNAs that are labeled in red fonts are oncomirs upregulated in colon cancer; whereas miRNAs that labeled in green fonts are tumor suppressor miRNAs downregulated in colon cancer.

Apart from their role in cancer initiation and development, the expression profile of miRNAs has been suggested to have a potential prognostic significance in many types of cancers, in which miRNAs can exert their regulatory roles by directly targeting genes in the key steps of metastatic processes of cancer [7]. Indeed, several specific miRNAs have been demonstrated to correlate with cancer metastasis by directly modulating gene expression in signaling networks (Figure 1). For instance, miR-200c plays an important role in mediating epithelial to mesenchymal transition (EMT) and the metastatic behavior in colon cancer by targeting gene expression involving in EMT pathways [8]. In this paper, we will discuss the role of miRNAs in the development and progression of colon cancer, focusing on their regulatory roles in signaling networks involving in metastatic colon cancer.

## 2. miRNAs in the Metastatic Procession of Colon Cancer

To date, a series of studies have examined miRNAs expression patterns in colon cancer using a variety of techniques including global miRNA expression profiling with deep sequencing and miRNA microarrays. These studies test the selected miRNAs with quantitative reverse transcriptase polymerase chain reaction and confirmed that miRNA expression of colon cancer is distinctly different than adjacent normal tissues. The consistently altered miRNA expression may have a role in the colon cancer development.

Michael and colleagues were the first team to find that miRNA expression patterns were altered in colon cancer. They showed that miR-143 and miR-145 were reduced in colon cancer and suggested these miRNAs were tumor suppressors. The original studies report that miRNAs have been found such that the expression of some miRNAs is suppressed in colon cancer; however, the expression of majority of identified miRNAs is globally elevated in this disease. A recent review on the profiling of miRNAs in colon cancer summarized that approximately 2/3 of the 164 altered miRNAs were elevated in tumors. It suggested that the miRNA processing machinery was not compromised in colon cancer. A series of studies reported that miR-21 is highly relevant in colon cancer and has important roles in cancer progression and metastasis. Other miRNAs which have been found to be altered in colon cancer which include miR-29a, the miR-17-92 cluster, miR-221, miR-222, miR-181b, miR-31, miR-183, miR-135, and the miR-200a/b/c family [9].

The progression of colon cancer metastasis is a complex process, including angiogenesis, migration, and invasion. The invasion ultimately leads to the intravasation and fluid transportation of cancer cells through circulatory or lymphatic system and extravasation to distant tissues where they eventually colonize and develop tumors. An increasing number of miRNAs has been identified to regulate signaling pathways associated with the process of colon cancer metastasis (Table 1) [10]. Angiogenesis is an essential step for the growth of both primary and metastatic tumors with bloodstream. Several lines of evidence have demonstrated that miRNAs may exert either a proangiogenic or an antiangiogenic effect in angiogenesis [11]. For examples, miR-221 and miR-222, whose expressions are related to the TNM stage and local invasion of cancer and are frequently elevated in colon cancer, were able to inhibit angiogenic activities in HUVEC (human umbilical vein endothelial cells) by directly targeting angiogenic genes of c-Kit (v-kit Hardy-Zuckerman 4 feline sarcoma viral oncogene homolog), Stat5A (signal transducer and activator of transcription 5A), ENOS (endothelial nitric oxide synthase), and ETS1 (v-ets erythroblastosis virus E26 oncogene homolog 1) [12]. miR-497 is downregulated in colon cancer, which is capable of inhibiting cancer cell survival, proliferation, and invasion [13], by targeting IGF1R (insulin-like growth factor 1 receptor), an angiogenic activator that contributes to angiogenesis in tumors [14]. Conversely, other miRNAs, such as miR-194, are also downregulated in colon cancer, which can directly target an inhibitor of angiogenesis by binding to the 3′ UTR of THBS1 mRNA that encodes thrombospondin-1 (TSP-1). These studies suggest that miRNAs may play a paradoxical role in tumor angiogenesis through regulating the expression of inhibitors or activators of angiogenesis [7].

In the progress of metastasis, cancer cells migrate and invade into adjacent tissues. Several miRNAs including miR-29a, miR-31, miR-103, and miR-107 have been identified to exert effects on the invasion of colon cancer cells* in vitro* and* in vivo* [15]. The expression of miR-103 and miR-107 were upregulated in colon cancer cells according to a recent study by Chen et al. [16], both of them were able to directly modulate the expression of DAPK1 (death-associated protein kinase 1) and KLF4 (Krüppel-like factor 4), and sequentially led an increased cell motility and suppression of cell-cell adhesion. This notion was also supported in the late study using murine model, in which an aberrant expression of miR-103/107 could enhance the invasion and liver metastasis in colon cancer through a mechanism of inhibiting the expression of DAPK and KLF4, by which KLF4 can influence cell cycle arrest [16]. Such an inhibitory role of miRNA in KLF4 expression of colon cancer was also found in miR-29a, of which miR-29 was upregulated in colon cancer tissues relate to normal mucosa [17]. In addition, more abundant miR-29a transcript could be detected in colon cancer with liver metastasis as compared to nonmetastatic cancer; it thus was suggested as a sensitive and potential marker for colon cancer metastasis [18]. miR-132 and miR-335 have been reported to inhibit colon cancer invasion and metastasis* via* directly targeting ZEB2 [19]. miR-592 and miR-552 were both overexpressed in primary colon cancer; Kim et al. found miR-592 and miR-552 could distinguish metastases in the lung between primary lung adenocarcinoma and colorectal cancer [20]. Sun et al. also found that miR-429, a member of miR-200 family, was significantly downregulated in colon cancer tissues and cell lines. Their further studies determined that miR-429 could inhibit the cell growth and invasion and regulate the expression of EMT-related marker genes by targeting Onecut2 in colon cancer [21].

Despite the exact mechanism of cancer cell intravasation into blood/lymph vessels by passing through the basement membrane and extravasation to a distant site far from the primary colon cancer remains elusive, it has been suggested that the immune system can cause degradation of cancer cells when they enter the bloodstream. There are a number of miRNAs that have been found to play critical roles in modulation of T and B lymphocyte activation, innate and adaptive immune responses, and the intravasation of colon cancer cells [22]. In this regard, miRNAs are suggested to be able to help colon cancer cells evade recognition by the immune system in the blood/lymph vessels, by which colon cancer cells escape from capillaries to invade the distant organ, a step before cancerous cell colonization [23]. miR-21 is a good example, which is more aberrantly expressed in metastatic colon cancer cells related to nonmetastatic cells and is related to lymph node metastasis. miR-21 can promote the intravasation of colon cancer cells by targeting tumor suppressor gene programmed cell death 4 (PDCD4) (Figure 1) [24].

The final step in metastasis is colonization of cancer cells from primary sites to distant tissues or organs. The circulating tumor cells in the bloodstream showing the affinity to particular sites are a hypothesis of seed and soil. The tumor cells are the “seed” and the specific organ microenvironment is the “soil” [16]. Metastatic colonization of this microenvironment may be dependent on the ability of cancer cells to proliferate and to adapt to new conditions. The establishment of distant metastases has been in part attributed to cancer stem cells (CSCs), a subpopulation of cancer cells that is characterized by the ability of self-renewal and multipotency of differentiation, which are believed to be responsible for resistances to chemo- and/or radio-therapy and the relapse of cancers [25]. It has been demonstrated that miRNAs are associated with the stemness of CSCs in colon cancers, and an impaired DICER1 function has recently been found to promote the stemness and metastasis in colon cancers. It is reported that miR-26b was downregulated in colon cancer LoVo cells, elevated expression of miR-26b could significantly reduce the capacity of cell proliferation and induce cell apoptosis in LoVo cells [26], suggesting that miRNAs may play a crucial role in colon cancer metastasis by altering properties of CSCs. miR-21 is an extensively studied oncogene capable of targeting multiple tumor suppressor genes, which is upregulated in chemoresistant colon cancer stem cells. An overexpression of miR-21 in HCT-116 cells led to the downregulation of PDCD4 and activation of Wnt/*β*-catenin signaling, along with an increased sphere forming ability* in vitro* and tumor formation in SCID mice, suggesting that miR-21 may play a key role in regulating stemness in colon cancer cells [27]. Recently, Ju et al. identified a subset of CD44^+^ HCT-15 and HCT-116 human colon cancer cells as CSCs of CRCs and found a significant upregulation of the protein Snail with a downregulation of miR-203. Interestingly, silencing miR-203 expression HCT-116 cells led to an increase of the stemness of colon CSCs, indicating that miR-203 may be an inhibitor of stemness in colon cancer stem cells [28].

Both of EMT and mesenchymal-to-epithelial transition (MET) are well-established biological events that play a role in the tissue homeostasis and pathogenesis in the colorectal carcinogenesis and cancer invasion [29]. One mission critical step in the metastatic cascade in cancers is the process of EMT. EMT is an evolutionarily conserved program of gene expression during which epithelial cells adopt characteristics of mesenchymal cells. The EMT is often activated during cancer progression and may promote resistance to therapy, which is regulated by a variety of signaling pathways, including transforming growth factor-beta (TGF*β*), hepatocyte growth factor (HGF), platelet derived growth factor (PDGF), and epidermal growth factor (EGF). Studies on miRNA expression patterns have been conducted to identify miRNAs with possible roles in TGF*β*-induced EMT. It is reported that miR-21 and miR-31 facilitate TGF*β*-induced EMT by targeting T-lymphoma invasion and metastasis 1 (TIAM1), repressing its translation rather than inducing mRNA degradation [30]. miR-200 family has been demonstrated as a key inhibitor for EMT in many types of human cancers. In the pathogenesis of metastatic CRC, abundant miR-200c could be detected in liver metastasis tissues, and overexpression of miR-200c in colon cancer cell lines led to a reduced expression of its target genes ZEB1, ETS1 and FLT1, and EMT markers (E-cadherin and vimentin) [8]. Similarly, the transcription factor AP4 plays a role in EMT, which is downregulated by DNA damage in a p53-dependent manner in CRC. Tumor suppressive miRNAs miR-15a and miR-16-1 were able to directly target AP4 mRNA, induce MET, and inhibit colorectal cancer cell migration and invasion. Moreover, enforced expression of miR-15a/16-1 led to a suppression of lung metastasis [31]. Recently, miR-147 was found to be able to induce a MET and reverses EGFR inhibitor resistance in colon cancers. An overexpression of miR-147 in CRC cell lines could induce MET by increasing the expression of CDH1 and decreasing that of ZEB1, inhibit the invasion and motility of the cells, and cause a G1 arrest by upregulating p27 and downregulating cyclin D1, as well as reverse the chemoresistance of the colon cancer cell line HCT116 to EGFR inhibitor, gefitinib [32].

The circulating exosomal miRNAs and tumor cells can be used as noninvasively surrogate markers for the diagnosis, prognosis, and therapeutic effectiveness of metastatic colon cancers [33–35]. By accessing circulating tumor cells (CTCs) in the blood of patients, patients at high risk for developing metastasis of relapse of colon cancers may be predicted. However, since a reliable epithelial cell mark is not always available for CTCs in metastatic colon cancer cells, especially during epithelial-mesenchymal transition, an accurate detection of CTCs may currently be a problem [36]. The emergence of circulating/exosomal miRNAs in the plasma of patients with colon cancers has thus used as biomarkers [34]. In this regard, Ogata-Kawata et al. recently identified seven miRNAs (let-7a, miR-1229, miR-1246, miR-150, miR-21, miR-223, and miR-23a) were significantly upregulated in the sera of primary CRC patients, and these miRNAs could be significantly downregulated after surgical resection of tumors [34]. Similarly, Slaby et al. analyzed the miRNAs in serum of presurgical patients with early stage CRCs and found a panel of six miRNAs (miR-15a, miR-103, miR-148a, miR-320a, miR-451, and miR-596) could be used as predictive markers for the risk of disease recurrence of early stage of CRCs [35]. In addition, the circulating/exosomal miRNAs can also be used as potential predictive markers for a therapeutic outcome [33]. By analysis of the expression of 742 miRNAs in plasma of metastatic CRC patients before onset and after four cycles of 5-fluorouracil (5-FU)/oxaliplatin, Kjersem et al. identified three miRNAs (miR-106a, miR-484, and miR-130b) that were significantly upregulated in nonresponders, and high levels of miR-27b, miR-148a, and miR-326 in plasma were associated with decreased progression-free survival [33].

## 3. miRNAs Regulate Metastatic Colon Cancer by Targeting Signaling Networks

Accumulating evidence has indicated that a number of miRNAs are capable of regulating tumor metastasis by modulating the expression of certain genes involved in signaling networks, including phosphatase and tensin homolog (PTEN)/phosphatidylinositol-3-kinase (PI3K), EGF receptor (EGFR), TGF*β*, and p53 pathways in colon cancers (Figure 1) (Table 2) [37]. For instances, PTEN is a phosphatase related to the PI3K pathway, which is involved in angiogenesis of tumors mainly through the PI3K pathway, mainly, despite some phosphatase-independent functions of PTEN were also demonstrated [38]. Dews et al. discovered that the miR-17-92 cluster could also mediate MYC-dependent tumor promoting effects by suppressing the expression of TSP-1 and CTGF (connective tissue growth factor), which are antiangiogenic factors [39]. Several miRNAs have been identified for targeting PETN/PI3K pathway, miR-17-92 cluster, also known as oncomir-1; one of its targets is PTEN, which is able to target PTEN and promote chemotherapeutic drug resistance and metastasis in colon cancers [40]. miR-32 was also identified to PTEN expression and promotes the growth, migration, and invasion of colorectal cancer cells [41]. miR-126 is another example, which is downregulated in primary colon cancer. The miR-126 can activate vascular endothelial to growth factor (VEGF) pathway by modulating the expression of sprouty-related protein SPRED1 and PIK3R2 (PI3K regulatory subunit 2), and mice knockdown of miR-126 exhibit phenotypes including a loss of vascular integrity and an inhibition of endothelial cell migration and angiogenesis [30]. In addition, miR-126 is also able to bind to the 3′ UTR of p85*β* (phosphatidylinositol-3-kinase regulatory subunit beta, PI3K*β*) mRNA and modulates its expression. PI3K*β* is a regulatory subunit involved in stabilization and propagation of PI3K pathway [42]. Apart from its regulatory role in PI3K pathway, the miR-126 was recently found to exert a role of tumor suppressor by inhibiting RhoA/ROCK signaling pathway through a mechanism of repressing RhoA expression. The activity of ROCK is involved in the invasion and metastasis of tumor cells including colon cancer, in which ROCK is the main RhoA downstream effector [42, 43].

EGFR is a receptor of tyrosine kinase (RTK) that plays a critical role in the survival, proliferation, migration, angiogenesis, and apoptosis [44]. Dysregulation of EGFR signaling as a consequence of overexpression, amplification, and mutation of the EGFR gene occurs frequently in several types of epithelial cancers, such as lung cancer and colon cancer. The emerging role of EGFR signaling in cancers has led the development of anti-EGFR agents, including tyrosine kinase inhibitors (TKIs) and monoclonal antibodies against EGFR [45]. However, most anti-EGFR targeted agents are able to frequently drug-resistance. Increasing numbers of evidence indicates that miRNAs are correlated with the drug resistance to anti-EGFR agents. In this context, miRNAs regulate the EGFR signaling;* vice versa*, EGFR signaling has an impact on the miRNA profiling of cancer cells [44, 46]. For example, miR-7 is a tumor suppressor in malignancies including CRC, which is able to target EGFR. A downregulation of miR-7 is an independent prognostic factor for poor survival. A combination of miR-7 and cetuximab, a monoclonal antibody against EGFR could enhance the growth inhibitory effect of each agent alone [47]. The miRNAs that target EGFR signaling pathway in colon cancers have been well documented [45, 48]. In a miRNA profiling analysis in metastatic colorectal cancer (mCRC) patients treated with anti-EGFR monoclonal antibodies conducted by Cappuzzo et al. using 183 mCRC cases, the authors identified miRNA cluster let-7c/miR-99a/miR-125b as a signature associated with an outcome different from that of anti-EGFR therapies, and this miRNA cluster may be used for the selection of patients with KRAS wild-type mCRC as good candidates for anti-EGFR therapy [49].

let-7 could downregulate KRAS with anticancer effects in the presence of activating KRAS mutations, a higher let-7a levels were significantly associated with better survival outcomes in patients who were KRAS-mutated colorectal cancer and underwent third-line therapy with cetuximab (an anti-EGFR monoclonal antibody) plus irinotecan, suggesting that let-7 may restore anti-EGFR therapy effects in patients with chemotherapy-refractory metastatic disease [50]. Other miRNAs, such as miR-181a [51] and miR-31-3p [52], have also been demonstrated as candidate markers for prediction of therapeutic responses to anti-EGFR agents in mCRC patients carrying wild-type KRAS. In addition, miR-181a was the most elevated in CRC with liver metastases and correlated with advanced stage, distant metastasis, which could serve as an independent prognostic factor of poor overall survival for patients with CRC. CRC cells transfected with miR-181a showed enhanced abilities of motility, invasion, tumor growth, and liver metastasis. Conversely, silencing the expression of miR-181a led to a reduced capacity for cell migration and invasion. Mechanistically, miR-181a could directly and functionally target Wnt inhibitor factor-1 (WIF-1) and suppress the expression of epithelial markers E-cadherin and *β*-catenin, while increase the expression of mesenchymal markers vimentin [53].

TGF*β*/Smad signaling is an important molecular pathway in the development, progression, and metastasis of colon cancer. miRNAs are important regulators in controlling the TGF*β* signaling pathway [54]. miR-130a/301a/454 family is upregulated in colon cancer tissues, which is predicted to target Smad4. An overexpression of miR-130a/301a/454 mimics could enhance cell proliferation and migration of HCT116 and SW480 colon cancer cells, while an inhibition of these miRNAs decreased the cell survival. TGF signaling plays a pivotal role in EMT of cancers. Recently, miRNAs have been suggested to be involved in the acquisition of acquiring stem-cell-like properties for cancer cells by regulating EMT signaling. For example, Hur et al. found that miR-200c was aberrantly expressed in metastatic colon tumor tissues and colon cancer cells, and this upregulated miR-200c was correlated with an reduction of the expression of its target genes: zinc finger E-box binding homeobox 1 (ZEB1), ETS1, and FMS-related tyrosine kinase 1 (FLT1), which in turn upregulates E-cadherin and downregulate the expression of vimentin, sequentially led an activation of EMT signaling pathway (Figure 1) [8, 55]. This observation was in line with a study by Korpal et al., in which the authors demonstrated that the effect of down- or upregulation of miR-200 family members caused a downstream increase/decrease of expression of ZEB1 and ZEB2 and then modulated the EMT pathway [55]. These studies demonstrate that miRNAs may play an important role in mediating EMT and metastatic behavior in the colon cancer.

The p53 protein is encoded by TP53 gene, which is one of the most important tumor suppressors that frequently inactivated in gastrointestinal cancer. miRNAs have recently been recognized as mediators and regulators of p53 signaling; vice versa, p53 can induce the expression and/or maturation of several miRNAs, including let-7a, miR-133a, and miR-16 in colon cancer cells [56]. Similarly, a downregulation of miR-145 and miR-34a was also found in colon cancer [25, 57]. miR-145 can act as a suppressor of tumor by inhibiting activities of KRAS and BRAF [57], while miR-34a may play a role as a tumor suppressor by regulating the Sirtuin 1- (SIRT1-) p53 pathway. In this context, miR-34a and p53 signaling can form a positive feedback loop, and the miR-34a inhibits the expression of SIRT1 [25, 58].

Wnt signaling also is an important pathway in the colon carcinogenesis. In this context, mutations of adenomatous polyposis coli (APC) occur in more than 60% of colon cancers, which leads to an activation of canonical Wnt/*β*-catenin signaling. The canonical Wnt pathway has been recognized to associate with early colon cancer development, suggesting that miRNAs correlated with regulation of Wnt signaling may play a role in colon cancer formation. Indeed, miR-135 was found to be upregulated in colon tumors and correlated with low level of APC, which could exert an effect on colon cancer* via* regulating Wnt/*β*-catenin signaling pathway in colon cancer [43].

miR-143 is a well-defined miRNA that is associated with colon cancer metastasis. It was downregulated in colon cancer and liver metastasis, and a less abundant miR-143 was found to associate with larger tumor size and longer disease-free interval in colon cancer, and an enhanced expression of miR-143 attenuates migration and invasion in colon cancer [59]. Mechanistically, miR-143 was identified to target metastasis-associated in colon cancer-1 (MACC1), a novel prognostic biomarker for metastasis occurrence, which was overexpressed in colon cancer and other cancer types [59]. Therefore, a downregulation of miR-143 could enhance colon cancer metastasis through a mechanism of MACC1-induced HGF-MET signaling pathway [60, 61].

## 4. miRNAs as Therapeutic Targets for Colon Cancer

In spite of the surgical resection and chemotherapy are highly effective for patients with colon cancer; tumor recurrence and chemoresistance remain the main challenge in colon cancer therapy. Increasing number of studies has demonstrated that miRNAs may serve as novel prognostic markers for prediction of chemotherapeutic responses and prognosis in patients with colon cancer (Table 3) [7]. In addition, alterations of miRNAs expression profiling are easily accessed in several tissues, such as blood, feces, and urine. It is widely known that a single miRNA can mediate the expression of several genes.* Vice versa*, an interest gene can be regulated by several miRNAs [62]. Therefore, miRNAs can be employed as potential targets and/or agents in anticancer therapy. Indeed, there are several miRNAs, including miR-140, miR-215, miR-224, and miR-20a, which have displayed a therapeutic effect in colon cancer [63, 64]. Recently, Li et al. reported that miR-34a is involved in sensitivity to 5-FU in part through its effects on lactate dehydrogenase A (LDHA) expression [65]; the expression of miR-520g is correlated with the reduced effectiveness of 5-FU in the inhibition of tumor growth in a mouse xenograft model. Further studies indicated that miR-520g mediated drug resistance through a downregulation of p21 expression, and the p53 could suppress miR-520g expression, which indicated that the p53/miR-520g/p21 signaling axis had an important role in the response of colon cancer to chemotherapy [66].

On the other hand, several miRNAs have recently been found as prognostic markers for colon cancer. For example, miR-29a is associated with TNM stages in colon cancer and related to colon cancer with liver metastasis [67]; an aberrant expression of miR-141 [68] and miR-18a [69] is associated with poor survival in colon cancer; and a downregulated expression of miR-320a and miR-498 is involved in shorter progression-free survival. Moreover, a recent study found that the expression of some miRNAs was associated with single nucleotide polymorphisms (SNPs) [70]. The function of miRNAs in modulating the expression of genes can be altered due to the presence of SNPs in pri-, pre-, and mature miRNAs, and these SNPs may be novel markers for diagnosis of colon cancer metastasis. For instance, SNPs in pre-miR-423 (rs6505162) and in pre-miR-608 (rs4919510) are largely associated with the recurrence-free survival in patients with colon cancer [70].

EGFR is a member of the human epidermal growth factor receptor or ErbB family of receptor tyrosine kinases. This trans-membrane glycoprotein may be activated through the binding of related ligands, which leads to EGFR forming homodimers or heterodimers with its family members such as ErbB2/neu, ErbB3/HER3, and ErbB4/HER4. This process can promote autophosphorylation of the intracellular domain through tyrosine kinase activity and stimulation of two major downstream signaling pathways, KRAS/RAF/ERK and PI3K/AKT. These pathways are all involved in cancer cell proliferation, invasion, metastasis, and neoangiogenesis. EGFR has been a therapeutic target in a range of tumors including colon cancer [48]. However, patients may have varied susceptibility to chemotherapeutic drugs of anti-EGFR agents, such as TKIs and monoclonal antibodies, implying the need for new biomarkers that are adopted to individual treatment. In this regard, KRAS mutation testing has thus become a routine in the clinic for EFGR targeting therapy, because anti-EGFR treatment is not efficient to KRAS-mutant tumors [71]. In addition, an aberrant expression miR-21 is demonstrated to correlate with a poor therapeutic survival in colon cancer patients received chemotherapy of 5-FU [72]. Mechanistically, miR-21 can target and inhibit hMSH2 and hMSH6 gene expression, the hMSH2 and hMSH6 are impairment of mismatch repair genes, a downregulation of them may lead to the resistance to 5-FU [73]. Other miRNAs also have been reported to be involved in chemoresponse to 5-FU. For instances, miR-143, miR-21, and miR-155 could regulate the expression of NF-*κ*B, an important transcription factor which involved in EGFR signaling pathway, sequentially enhance tumor apoptosis after exposure to 5-FU [74]; and the miR-181b and let-7g also have been widely involved in chemoresponse to S-1, a 5-FU-based antimetabolite [75].

Amongst the various miRNAs, the roles of miR-126, miR-328, and miR-497 in CRC have been extensively studied and the outcomes of these studies have shown high concordance. miR-126 is a potential tumor suppressor and is downregulated and correlated with the metastasis in CRC. The clinical role of miR-126 has been documented by Ebrahimi et al. [76]. miR-126 can target multiple signaling including PI3K/AKT/mTOR and Rho/ROCK pathways to facilitate the suppression of cancers, suggesting that it is a potential candidate for future therapeutic approaches in CRC [77]. miR-328 is another miRNA with therapeutic potential for CRC. miRNA microarray analysis of stem-like side population (SP) cells revealed that miR-328 was a potential stemness miRNA for SP phenotype in CRC [78]. miR-328 was significantly reduced in CRC SP cells relative to the non-SP cells; functional studies using a gain-loss approach further demonstrated that the abundance of miR-328 could affect the frequency of SP cells in CRC, and ectopic expression of miR-328 could reverse drug resistance and inhibit cell invasion of SP cells; mechanistically, miR-328 was able to directly target ATP-binding cassette subfamily G2 (ABCG2) and matrix metallopeptidase 16 (MMP16) [78]. In this context, ABCG2 is an ABC transporter; an inhibition of ABCG2 may reverse drug resistance of cancer cells, suggesting that miR-328 is a novel therapeutic approach for antimetastatic therapy [18]. Similarly, miRNA-497 can target insulin-like growth factor 1 receptor (IGF1-R) to repress the tumor growth, which is downregulated and associated with the cell survival, proliferation and invasion, and the sensitivity to chemotherapeutic drugs cisplatin and 5-FU in human CRC [13]. Recently, Qiu et al. found that a combination of miR-497 and bufalin had a synergistic effect on the inhibition of colorectal cancer metastasis in a nude mouse model, suggesting a potential of miR-497 in clinical therapy for CRC metastasis [79].

## 5. Perspectives and Challenges

miRNAs are a class of regulators at the posttranscription level; they exhibit different expression patterns in various types of cancers. The dysregulation of miRNAs has been demonstrated to play a significantly effect on cell invasion, metastasis, and drug resistance in colon cancer through the interaction of the intracellular signaling networks. In addition, an expanding list of dysregulated miRNAs has been explored as potential biomarkers for the diagnosis and prognosis of colon cancer. The alteration of miRNA expression profile can be determined in colon cancer tissues and in circulating specimens. In the therapeutic standpoint, miRNAs are mainly involved in anticancer therapy by restoring tumor suppressor genes or inhibiting oncogenes [80]. They may enhance the sensitivity of tumor cells to chemotherapy or inhibit cell stemness [81]. In addition, a single miRNA is able to target multiple genes in one metabolic pathway.

Chemotherapy is a common treatment modality for advanced or metastatic colon cancer. However, patients have different sensitivities to chemotherapeutic drugs due to individual genetic background and tumor progression. Furthermore, chemotherapy has significant side effects in general. miRNA-based therapy may be an effective approach for the individual treatment of colon cancer. Unfortunately, the successful development of miRNA-based therapeutics for colon cancer has many challenging hurdles to overcome. For instance, the best miRNAs to be targeted in colon cancer are yet to be defined, which need a better understanding on the biology of how miRNAs affect colon cancer. Nevertheless, anti-miR-21-based therapies may provide useful strategies based on what is already known about the biology. On the other hand, the major issue is how to achieve effective delivery without causing unnecessary side effects. In addition, these strategies need to select the appropriate patient populations that may have therapeutic benefit. Since a single miRNA can modulate the expression of a number of genes, it is a challenge to understand the exact process of a candidate miRNA involved in the signaling networks of colon cancer. To date, researchers have started to uncover the complicated relationship between miRNAs and the signaling networks in colon cancer; these studies will improve our understanding of the underlying molecular mechanism of colon cancer [80, 82]. While there is much to be done, we are optimistic in using miRNA-based therapeutics to fight against colon cancer in the future.

## Figures and Tables

**Table 1:** The expressions of miRNAs and their corresponding target genes involved in metastatic colon cancer.

MicroRNA	Up/ down	Target gene(s)	References
let-7	Down	RAS, HMGA2	[83, 84]
miR-101b	Down	COX2	[85]
miR-124a	Down	CDK6, Rb	[86]
miR-133b	Down	c-Met, K-Ras, TAp63	[87]
miR-137	Down	Cdc42, LSD-1, TGF2I	[88]
miR-192	Down	DHFR, p53	[89]
miR-212		MnSOD	[90]
miR-27a	Down	SGPP1, Smad2	[91]
miR-214	Down	TP53, *β*-catenin, TGFR2, BAX, CDKN2b, EGFR, TFAP2C	[23]
miR-449b	Down	E2F3, CCND1	[61]
miR-497	Down	IGF1R	[13]
miR-103	Up	DAPK, KLF4	[16]
miR-107	Up	DAPK, KLF4	[16]
miR-122	Up	CAT1, ADAM17, cyclin-G, Bcl-W	[92, 93]
miR-155	Up	MSH2, MSH6, MCH1, AKT	[74, 94]
miR-182	Up	ENTPD5, IGFR1, FoxF2	[95, 96]
miR-210	Up	K-Ras, Bcl-2	[12, 97]
miR-221	Up	c-Kit, Stat5A, ETS1, ENOS	[12, 18]
miR-451	Up	MIF, IL6R	[98]
miR-675	Up	RB	[99]

**Table 2:** Key signal pathways in metastatic colon cancer targeted by microRNAs.

MicroRNA	Signal pathway	References
miR-141	EMT	[68]
miR-15a	EMT	[31]
miR-16-1	EMT	[31]
miR-200c	EMT	[8]
let-7	EMT	[100]
miR-126	RhoA/ROCK, VEGF, and PI3K-AKT	[37, 42]
miR-135b	Wnt/*β*-catenin	[101, 102]
miR-143	HGF/MET and EGFR	[57]
miR-144	mTOR	[103]
miR-145	EGFR	[59]
miR-17-92	PI3K	[18]
miR-18a	Autophagy pathway	[69]
miR-185	HIF-2*α*	[104]
miR-196a	AKT	[105]
miR-21	Autophagy and stemness pathways	[27, 66]
miR-34a	Fra-1, E2F, and SIRT1-p53	[106]
miR-18	EGFR and KRAS	[107]
miR-30a	PI3K	[108]
miR-32	PTEN	[41]
miR-129	KRAS	[109]
miR-125b	p53	[110]
miR-155	PTEN	[111]
miR-203	EGFR and AKT	[112]
miR-223	PTEN	[113]
miR-525	PI3K	[114]

**Table 3:** MicroRNAs as biomarkers and therapeutic targets in metastatic colon cancer.

MicroRNA	Metastatic site	Prognosis	References
miR-10b	Liver		[95]
miR-101b	Liver		[85]
miR-103	Liver		[16]
miR-107	Liver		[16]
miR-122	Liver		[93]
miR-143	Liver	Poor	[59]
miR-144	Liver	Poor	[103]
miR-15a	Lung		[31]
miR-16-1	Lung		[31]
miR-185	Lymph node		[104]
miR-195	Lymph node	Poor	[115]
miR-200c	Liver and lymph node	Poor	[8, 116]
miR-21	Liver and lymph node	Poor	[30, 74, 90]
miR-22	Liver		[117]
miR-29a	Liver	Poor	[67, 118]
miR-34a	Lymph node		[106]
miR-320a	Liver	Poor	[119]
miR-592	Lymph node		[20]
miR-493	Liver		[120, 121]
miR-499-5p	Lymph node and liver		[122]
miR-574-5p	Liver		[123]

## Abbreviations

ABCG2: ATP-binding cassette, subfamily G (WHITE), member 2
ADAM17: ADAM metallopeptidase domain 17
AKT: v-akt murine thymoma viral oncogene homolog
Bcl-W: B-cell lymphoma 2 like 2
BAX: BCL2-associated X protein
CAT1: Cationic amino acid transporter 1
CCND1: Cyclin D1
Cdc42: Cell division cycle 42
CDK6: Cyclin-dependent kinase 6
CDKN: Cyclin-dependent kinase inhibitor
c-Kit: v-kit Hardy-Zuckerman 4 feline sarcoma viral oncogene homolog
c-Met: Hepatocyte growth factor receptor
COX2: Cytochrome c oxidase subunit II
DAPK: Death-associated protein kinase
DHFR: Dihydrofolate reductase
E2F: Elongation 2 factor
EGFR: Epidermal growth factor receptor
EMT: Epithelial to mesenchymal transition
ENOS: Endothelial nitric oxide synthase
ENTPD: Ectonucleoside triphosphate diphosphohydrolase
ETS: v-ets erythroblastosis virus E26 oncogene homolog
FIH-1: Hypoxia inducible factor 1, alpha subunit inhibitor
HGF: Hepatocyte growth factor
HIF-2*α*: Hypoxia inducible factor-2*α*
HMGA2: High mobility group A2
IGF1R: Insulin-like growth factor 1 receptor
KLF4: Krüppel-like factor 4
K-Ras: Kirsten rat sarcoma viral oncogene homolog
LSD-1: Lysine(K)-specific demethylase 1
MAPK: Mitogen-activated protein kinase
MCH1: Melanin-concentrating hormone receptor 1
MET: Mesenchymal to epithelial transition
MIF: Macrophage migration inhibitory factor
MMP16: Matrix metallopeptidase 16
MnSOD: Manganese superoxide dismutase
MSH: mutS homolog
mTOR: Mammalian target of rapamycin
PI3K: Phosphatidylinositol-3-kinase
Rb: Retinoblastoma protein
RhoA: RAS homolog family member A
ROCK: Rho-associated, coiled-coil containing protein kinase
SGPP1: Sphingosine-1-phosphate phosphatase 1
SIRT1: Sirtuin 1
Smad2: SMAD family member 2
Stat5A: Signal transducer and activator of transcription 5A
TGFR2: Transforming growth factor, beta receptor II
TFAP2C: Transcription factor AP-2 gamma
THBS1: Thrombospondin 1
TIAM1: T-cell lymphoma invasion and metastasis 1
VCAM1: Vascular cell adhesion molecule 1
VEGF: Vascular endothelial to growth factor.

## References

[1] Ferlay J., Shin H.-R., Bray F., Forman D., Mathers C., Parkin D. M. (2010). Estimates of worldwide burden of cancer in 2008: GLOBOCAN 2008. *International Journal of Cancer*.

[2] Fritzmann J., Morkel M., Besser D. (2009). A colorectal cancer expression profile that includes transforming growth factor *β* inhibitor BAMBI predicts metastatic potential. *Gastroenterology*.

[3] Han J., Lee Y., Yeom K.-H., Kim Y.-K., Jin H., Kim V. N. (2004). The Drosha-DGCR8 complex in primary microRNA processing. *Genes and Development*.

[4] Manikandan J., Aarthi J. J., Kumar S. D., Pushparaj P. N. (2008). Oncomirs: the potential role of non-coding microRNAs in understanding cancer. *Bioinformation*.

[5] Schee K., Fodstad Ø., Flatmark K. (2010). MicroRNAs as biomarkers in colorectal cancer. *The American Journal of Pathology*.

[6] di Leva G., Garofalo M., Croce C. M. (2014). MicroRNAs in cancer. *Annual Review of Pathology*.

[7] Muhammad S., Kaur K., Huang R. (2014). MicroRNAs in colorectal cancer: role in metastasis and clinical perspectives. *World Journal of Gastroenterology*.

[8] Hur K., Toiyama Y., Takahashi M. (2013). MicroRNA-200c modulates epithelial-tomesenchymal transition (EMT) in human colorectal cancer metastasis. *Gut*.

[9] Hong L., Han Y., Yang J. (2014). MicroRNAs in gastrointestinal cancer: prognostic significance and potential role in chemoresistance. *Expert Opinion on Biological Therapy*.

[10] Hanahan D., Weinberg R. A. (2011). Hallmarks of cancer: the next generation. *Cell*.

[11] Bonauer A., Carmona G., Iwasaki M. (2009). MicroRNA-92a controls angiogenesis and functional recovery of ischemic tissues in mice. *Science*.

[12] Ota T., Doi K., Fujimoto T. (2012). KRAS up-regulates the expression of miR-181a, miR-200c and miR-210 in a three-dimensional-specific manner in DLD-1 colorectal cancer cells. *Anticancer Research*.

[13] Guo S. T., Jiang C. C., Wang G. P. (2013). MicroRNA-497 targets insulin-like growth factor 1 receptor and has a tumour suppressive role in human colorectal cancer. *Oncogene*.

[14] Dobrucki L. W., Tsutsumi Y., Kalinowski L. (2010). Analysis of angiogenesis induced by local IGF-1 expression after myocardial infarction using microSPECT-CT imaging. *Journal of Molecular and Cellular Cardiology*.

[15] Chang K. H., Miller N., Kheirelseid E. A. H. (2011). MicroRNA signature analysis in colorectal cancer: identification of expression profiles in stage II tumors associated with aggressive disease. *International Journal of Colorectal Disease*.

[16] Chen H.-Y., Lin Y.-M., Chung H.-C. (2012). MiR-103/107 promote metastasis of colorectal cancer by targeting the metastasis suppressors DAPK and KLF4. *Cancer Research*.

[17] Fu J., Tang W., Du P. (2012). Identifying MicroRNA-mRNA regulatory network in colorectal cancer by a combination of expression profile and bioinformatics analysis. *BMC Systems Biology*.

[18] Tokarz P., Blasiak J. (2012). The role of microRNA in metastatic colorectal cancer and its significance in cancer prognosis and treatment. *Acta Biochimica Polonica*.

[19] Sun Z., Zhang Z., Liu Z., Qiu B., Liu K., Dong G. (2014). MicroRNA-335 inhibits invasion and metastasis of colorectal cancer by targeting ZEB2. *Medical Oncology*.

[20] Kim J., Lim N. J., Jang S. G., Kim H. K., Lee G. K. (2014). miR-592 and miR-552 can distinguish between primary lung adenocarcinoma and colorectal cancer metastases in the lung. *Anticancer Research*.

[21] Sun Y., Shen S., Liu X. (2014). MiR-429 inhibits cells growth and invasion and regulates EMT-related marker genes by targeting Onecut2 in colorectal carcinoma. *Molecular and Cellular Biochemistry*.

[22] Lu L.-F., Liston A. (2009). MicroRNA in the immune system, microRNA as an immune system. *Immunology*.

[23] Penna E., Orso F., Cimino D. (2011). MicroRNA-214 contributes to melanoma tumour progression through suppression of TFAP2C. *EMBO Journal*.

[24] Vickers M. M., Bar J., Gorn-Hondermann I. (2012). Stage-dependent differential expression of microRNAs in colorectal cancer: potential role as markers of metastatic disease. *Clinical and Experimental Metastasis*.

[25] Hudson J. D., Shoaibi M. A., Maestro R., Carnero A., Hannon G. J., Beach D. H. (1999). A proinflammatory cytokine inhibits p53 tumor suppressor activity. *Journal of Experimental Medicine*.

[26] Ma Y.-L., Zhang P., Wang F. (2011). Human embryonic stem cells and metastatic colorectal cancer cells shared the common endogenous human microRNA-26b. *Journal of Cellular and Molecular Medicine*.

[27] Yu Y., Kanwar S. S., Patel B. B. (2012). MicroRNA-21 induces stemness by downregulating transforming growth factor beta receptor 2 (TGFbetaR2) in colon cancer cells. *Carcinogenesis*.

[28] Ju S.-Y., Chiou S.-H., Su Y. (2014). Maintenance of the stemness in CD44^+^ HCT-15 and HCT-116 human colon cancer cells requires miR-203 suppression. *Stem Cell Research*.

[29] Sipos F., Galamb O. (2012). Epithelial-to-mesenchymal and mesenchymal-to-epithelial transitions in the colon. *World Journal of Gastroenterology*.

[30] Cottonham C. L., Kaneko S., Xu L. (2010). miR-21 and miR-31 converge on TIAM1 to regulate migration and invasion of colon carcinoma cells. *The Journal of Biological Chemistry*.

[31] Shi L., Jackstadt R., Siemens H., Li H., Kirchner T., Hermeking H. (2014). P53-induced miR-15a/16-1 and AP4 form a double-negative feedback loop to regulate epithelial-mesenchymal transition and metastasis in colorectal cancer. *Cancer Research*.

[32] Lee C. G., McCarthy S., Gruidl M., Timme C., Yeatman T. J. (2014). MicroRNA-147 induces a mesenchymal-to-epithelial transition (MET) and reverses EGFR inhibitor resistance. *PLoS ONE*.

[33] Kjersem J. B., Ikdahl T., Lingjaerde O. C., Guren T., Tveit K. M., Kure E. H. (2014). Plasma microRNAs predicting clinical outcome in metastatic colorectal cancer patients receiving first-line oxaliplatin-based treatment. *Molecular Oncology*.

[34] Ogata-Kawata H., Izumiya M., Kurioka D. (2014). Circulating exosomal microRNAs as biomarkers of colon cancer. *PLoS ONE*.

[35] Slaby O., Svoboda M., Michalek J., Vyzula R., Cho W. C. (2011). MicroRNAs in colorectal cancer. *MicroRNAs in Cancer Translational Research*.

[36] Kantara C., O'Connell M. R., Luthra G. (2014). Methods for detecting circulating cancer stem cells (CCSCs) as a novel approach for diagnosis of colon cancer relapse/metastasis. *Laboratory Investigation*.

[37] Fish J. E., Santoro M. M., Morton S. U. (2008). miR-126 regulates angiogenic signaling and vascular integrity. *Developmental Cell*.

[38] Rodriguez S., Huynh-Do U. (2012). The role of PTEN in tumor angiogenesis. *Journal of Oncology*.

[39] Dews M., Homayouni A., Yu D. (2006). Augmentation of tumor angiogenesis by a Myc-activated microRNA cluster. *Nature Genetics*.

[40] Fang L., Li H., Wang L. (2014). MicroRNA-17-5p promotes chemotherapeutic drug resistance and tumour metastasis of colorectal cancer by repressing PTEN expression. *Oncotarget*.

[41] Wu W., Yang J., Feng X. (2013). MicroRNA-32 (miR-32) regulates phosphatase and tensin homologue (PTEN) expression and promotes growth, migration, and invasion in colorectal carcinoma cells. *Molecular Cancer*.

[42] Li N., Tang A., Huang S. (2013). MiR-126 suppresses colon cancer cell proliferation and invasion via inhibiting RhoA/ROCK signaling pathway. *Molecular and Cellular Biochemistry*.

[43] Faber C., Kirchner T., Hlubek F. (2009). The impact of microRNAs on colorectal cancer. *Virchows Archiv*.

[44] Gomez G. G., Wykosky J., Zanca C., Furnari F. B., Cavenee W. K. (2013). Therapeutic resistance in cancer: microRNA regulation of EGFR signaling networks. *Cancer Biology and Medicine*.

[45] Giampieri R., Scartozzi M., del Prete M. (2013). Molecular biomarkers of resistance to anti-EGFR treatment in metastatic colorectal cancer, from classical to innovation. *Critical Reviews in Oncology/Hematology*.

[46] Deyati A., Bagewadi S., Senger P., Hofmann-Apitius M., Novac N. (2015). Systems approach for the selection of micro-RNAs as therapeutic biomarkers of anti-EGFR monoclonal antibody treatment in colorectal cancer. *Scientific Reports*.

[47] Suto T., Yokobori T., Yajima R. (2015). MicroRNA-7 expression in colorectal cancer is associated with poor prognosis and regulates cetuximab sensitivity via EGFR regulation. *Carcinogenesis*.

[48] Mlcochova J., Faltejskova P., Nemecek R., Svoboda M., Slaby O. (2013). MicroRNAs targeting EGFR signalling pathway in colorectal cancer. *Journal of Cancer Research and Clinical Oncology*.

[49] Cappuzzo F., Sacconi A., Landi L. (2014). MicroRNA signature in metastatic colorectal cancer patients treated with anti-EGFR monoclonal antibodies. *Clinical Colorectal Cancer*.

[50] Ruzzo A., Graziano F., Vincenzi B. (2012). High let-7a microRNA levels in *KRAS*-mutated colorectal carcinomas may rescue anti-EGFR therapy effects in patients with chemotherapy-refractory metastatic disease. *The Oncologist*.

[51] Pichler M., Winter E., Ress A. L. (2014). MiR-181a is associated with poor clinical outcome in patients with colorectal cancer treated with EGFR inhibitor. *Journal of Clinical Pathology*.

[52] Manceau G., Imbeaud S., Thiebaut R. (2014). Hsa-miR-31-3p expression is linked to progression-free survival in patients with KRAS wild-type metastatic colorectal cancer treated with anti-EGFR therapy. *Clinical Cancer Research*.

[53] Ji D., Chen Z., Li M. (2014). MicroRNA-181a promotes tumor growth and liver metastasis in colorectal cancer by targeting the tumor suppressor WIF-1. *Molecular Cancer*.

[54] Butz H., Rácz K., Hunyady L., Patócs A. (2012). Crosstalk between TGF-*β* signaling and the microRNA machinery. *Trends in Pharmacological Sciences*.

[55] Korpal M., Lee E. S., Hu G., Kang Y. (2008). The miR-200 family inhibits epithelial-mesenchymal transition and cancer cell migration by direct targeting of E-cadherin transcriptional repressors ZEB1 and ZEB2. *The Journal of Biological Chemistry*.

[56] Rokavec M., Li H., Jiang L. (2014). The p53/microRNA connection in gastrointestinal cancer. *Clinical and Experimental Gastroenterology*.

[57] Pagliuca A., Valvo C., Fabrizi E. (2013). Analysis of the combined action of miR-143 and miR-145 on oncogenic pathways in colorectal cancer cells reveals a coordinate program of gene repression. *Oncogene*.

[58] Bu P., Chen K.-Y., Chen J. H. (2013). A microRNA miR-34a-regulated bimodal switch targets notch in colon cancer stem cells. *Cell Stem Cell*.

[59] Pekow J., Meckel K., Dougherty U. (2015). Tumor suppressors miR-143 and miR-145 and predicted target proteins API5, ERK5, K-RAS, and IRS-1 are differentially expressed in proximal and distal colon. *The American Journal of Physiology—Gastrointestinal and Liver Physiology*.

[60] Arndt G. M., Dossey L., Cullen L. M. (2009). Characterization of global microRNA expression reveals oncogenic potential of miR-145 in metastatic colorectal cancer. *BMC Cancer*.

[61] Fang Y., Gu X., Li Z., Xiang J., Chen Z. (2013). miR-449b inhibits the proliferation of SW1116 colon cancer stem cells through downregulation of CCND1 and E2F3 expression. *Oncology Reports*.

[62] Niyazi M., Zehentmayr F., Niemöller O. M. (2011). MiRNA expression patterns predict survival in glioblastoma. *Radiation Oncology*.

[63] Mencia N., Selga E., Noé V., Ciudad C. J. (2011). Underexpression of miR-224 in methotrexate resistant human colon cancer cells. *Biochemical Pharmacology*.

[64] Song B., Wang Y., Titmus M. A. (2010). Molecular mechanism of chemoresistance by miR-215 in osteosarcoma and colon cancer cells. *Molecular Cancer*.

[65] Li X., Zhao H., Zhou X., Song L. (2014). Inhibition of lactate dehydrogenase A by microRNA-34a resensitizes colon cancer cells to 5-fluorouracil. *Molecular Medicine Reports*.

[66] Zhang Y., Geng L., Talmon G., Wang J. (2015). MicroRNA-520g confers drug resistance by regulating p21 expression in colorectal cancer. *The Journal of Biological Chemistry*.

[67] Weissmann-Brenner A., Kushnir M., Yanai G. L. (2012). Tumor microRNA-29a expression and the risk of recurrence in stage II colon cancer. *International Journal of Oncology*.

[68] Yin J., Bai Z., Song J. (2014). Differential expression of serum miR-126, miR-141 and miR-21 as novel biomarkers for early detection of liver metastasis in colorectal cancer. *Chinese Journal of Cancer Research*.

[69] Fujiya M., Konishi H., Kamel M. K. M. (2013). microRNA-18a induces apoptosis in colon cancer cells via the autophagolysosomal degradation of oncogenic heterogeneous nuclear ribonucleoprotein A1. *Oncogene*.

[70] Xing J., Wan S., Zhou F. (2012). Genetic polymorphisms in pre-microRNA genes as prognostic markers of colorectal cancer. *Cancer Epidemiology Biomarkers and Prevention*.

[71] Engstrom P. F., Arnoletti J. P., Benson A. B. (2009). NCCN clinical practice guidelines in oncology: colon cancer. *Journal of the National Comprehensive Cancer Network*.

[72] Schetter A. J., Leung S. Y., Sohn J. J. (2008). MicroRNA expression profiles associated with prognosis and therapeutic outcome in colon adenocarcinoma. *Journal of the American Medical Association*.

[73] Valeri N., Gasparini P., Braconi C. (2010). MicroRNA-21 induces resistance to 5-fluorouracil by down-regulating human DNA MutS homolog 2 (hMSH2). *Proceedings of the National Academy of Sciences of the United States of America*.

[74] Bakirtzi K., Hatziapostolou M., Karagiannides I. (2011). Neurotensin signaling activates microRNAs-21 and -155 and Akt, promotes tumor growth in mice, and is increased in human colon tumors. *Gastroenterology*.

[75] Nakajima G., Hayashi K., Xi Y. (2006). Non-coding microRNAs hsa-let-7g and hsa-miR-181b are associated with chemoresponse to S-1 in colon cancer. *Cancer Genomics and Proteomics*.

[76] Ebrahimi F., Gopalan V., Smith R. A., Lam A. K.-Y. (2014). MiR-126 in human cancers: clinical roles and current perspectives. *Experimental and Molecular Pathology*.

[77] Guo C., Sah J. F., Beard L., Willson J. K. V., Markowitz S. D., Guda K. (2008). The noncoding RNA, miR-126, suppresses the growth of neoplastic cells by targeting phosphatidylinositol 3-kinase signaling and is frequently lost in colon cancers. *Genes Chromosomes and Cancer*.

[78] Xu X. T., Xu Q., Tong J. L. (2012). MicroRNA expression profiling identifies miR-328 regulates cancer stem cell-like SP cells in colorectal cancer. *British Journal of Cancer*.

[79] Qiu Y.-Y., Hu Q., Tang Q.-F. (2014). MicroRNA-497 and bufalin act synergistically to inhibit colorectal cancer metastasis. *Tumor Biology*.

[80] Stiegelbauer V., Perakis S., Deutsch A., et al (2014). MicroRNAs as novel predictive biomarkers and therapeutic targets in colorectal cancer. *World Journal of Gastroenterology*.

[81] Yu H. W., Cho W. C. (2015). The emerging role of miRNAs in combined cancer therapy. *Expert Opinion on Biological Therapy*.

[82] Wang F., Wong S. C. C., Chan L. W. C., Cho W. C. S., Yip S. P., Yung B. Y. M. (2014). Multiple regression analysis of mRNA-miRNA associations in colorectal cancer pathway. *BioMed Research International*.

[83] Motoyama K., Inoue H., Nakamura Y., Uetake H., Sugihara K., Mori M. (2008). Clinical significance of high mobility group A2 in human gastric cancer and its relationship to let-7 MicroRNA family. *Clinical Cancer Research*.

[84] Johnson S. M., Grosshans H., Shingara J. (2005). RAS is regulated by the let-7 microRNA family. *Cell*.

[85] Strillacci A., Griffoni C., Sansone P. (2009). MiR-101 downregulation is involved in cyclooxygenase-2 overexpression in human colon cancer cells. *Experimental Cell Research*.

[86] Ueda Y., Ando T., Nanjo S., Ushijima T., Sugiyama T. (2014). DNA methylation of microRNA-124a is a potential risk marker of colitis-associated cancer in patients with ulcerative colitis. *Digestive Diseases and Sciences*.

[87] Lin C. W., Li X. R., Zhang Y. (2014). TAp63 suppress metastasis via miR-133b in colon cancer cells. *British Journal of Cancer*.

[88] Balaguer F., Link A., Lozano J. J. (2010). Epigenetic silencing of miR-137 is an early event in colorectal carcinogenesis. *Cancer Research*.

[89] Braun C. J., Zhang X., Savelyeva I. (2008). p53-responsive microRNAs 192 and 215 are capable of inducing cell cycle arrest. *Cancer Research*.

[90] Meng X., Wu J., Pan C. (2013). Genetic and epigenetic down-regulation of microRNA-212 promotes colorectal tumor metastasis via dysregulation of MnSOD. *Gastroenterology*.

[91] Bao Y., Chen Z., Guo Y. (2014). Tumor suppressor microRNA-27a in colorectal carcinogenesis and progression by targeting SGPP1 and Smad2. *PLoS ONE*.

[92] He J., Xie G., Tong J. (2014). Overexpression of microRNA-122 re-sensitizes 5-FU-resistant colon cancer cells to 5-FU through the inhibition of PKM2 in vitro and in vivo. *Cell Biochemistry and Biophysics*.

[93] Iino I., Kikuchi H., Miyazaki S. (2013). Effect of miR-122 and its target gene cationic amino acid transporter 1 on colorectal liver metastasis. *Cancer Science*.

[94] Rossi S., Di Narzo A. F., Mestdagh P. (2012). MicroRNAs in colon cancer: a roadmap for discovery. *FEBS Letters*.

[95] Pizzini S., Bisognin A., Mandruzzato S. (2013). Impact of microRNAs on regulatory networks and pathways in human colorectal carcinogenesis and development of metastasis. *BMC Genomics*.

[96] Zhang Y., Wang X., Wang Z., Tang H., Fan H., Guo Q. (2015). miR-182 promotes cell growth and invasion by targeting forkhead box F2 transcription factor in colorectal cancer. *Oncology Reports*.

[97] Sun Y., Xing X., Liu Q. (2015). Hypoxia-induced autophagy reduces radiosensitivity by the HIF-1alpha/miR-210/Bcl-2 pathway in colon cancer cells. *International Journal of Oncology*.

[98] Liu D., Liu C., Wang X., Ingvarsson S., Chen H. (2014). MicroRNA-451 suppresses tumor cell growth by down-regulating IL6R gene expression. *Cancer Epidemiology*.

[99] Tsang W. P., Ng E. K. O., Ng S. S. M. (2010). Oncofetal H19-derived miR-675 regulates tumor suppressor RB in human colorectal cancer. *Carcinogenesis*.

[100] Shell S., Park S.-M., Radjabi A. R. (2007). Let-7 expression defines two differentiation stages of cancer. *Proceedings of the National Academy of Sciences of the United States of America*.

[101] Khatri R., Subramanian S. (2013). MicroRNA-135b and its circuitry networks as potential therapeutic targets in colon cancer. *Frontiers in Oncology*.

[102] Valeri N., Braconi C., Gasparini P. (2014). MicroRNA-135b promotes cancer progression by acting as a downstream effector of oncogenic pathways in colon cancer. *Cancer Cell*.

[103] Iwaya T., Yokobori T., Nishida N. (2012). Downregulation of miR-144 is associated with colorectal cancer progression via activation of mTOR signaling pathway. *Carcinogenesis*.

[104] Lu Z. J., Lu L. G., Tao K. Z. (2014). MicroRNA-185 suppresses growth and invasion of colon cancer cells through inhibition of the hypoxiainducible factor-2alpha pathway in vitro and in vivo. *Molecular Medicine Reports*.

[105] Schimanski C. C., Frerichs K., Rahman F. (2009). High miR-196a levels promote the oncogenic phenotype of colorectal cancer cells. *World Journal of Gastroenterology*.

[106] Wu J., Wu G., Lv L. (2012). MicroRNA-34a inhibits migration and invasion of colon cancer cells via targeting to Fra-1. *Carcinogenesis*.

[107] Komatsu S., Ichikawa D., Takeshita H. (2014). Circulating miR-18a: a sensitive cancer screening biomarker in human cancer. *In Vivo*.

[108] Zhong M., Bian Z., Wu Z. (2013). MiR-30a suppresses cell migration and invasion through downregulation of PIK3CD in colorectal carcinoma. *Cellular Physiology and Biochemistry*.

[109] Wu J., Qian J., Li C. (2010). miR-129 regulates cell proliferation by downregulating Cdk6 expression. *Cell Cycle*.

[110] Banzhaf-Strathmann J., Edbauer D. (2014). Good guy or bad guy: the opposing roles of microRNA 125b in cancer. *Cell Communication and Signaling*.

[111] Li T., Yang J., Lv X. (2014). miR-155 regulates the proliferation and cell cycle of colorectal carcinoma cells by targeting E2F2. *Biotechnology Letters*.

[112] Li J., Chen Y., Zhao J., Kong F., Zhang Y. (2011). MiR-203 reverses chemoresistance in p53-mutated colon cancer cells through downregulation of Akt2 expression. *Cancer Letters*.

[113] Wu L., Li H., Jia C. Y. (2012). MicroRNA-223 regulates FOXO1 expression and cell proliferation. *FEBS Letters*.

[114] Arcaroli J. J., Quackenbush K. S., Powell R. W. (2012). Common PIK3CA mutants and a novel 3′ UTR mutation are associated with increased sensitivity to saracatinib. *Clinical Cancer Research*.

[115] Wang X., Wang J., Ma H., Zhang J., Zhou X. (2012). Downregulation of miR-195 correlates with lymph node metastasis and poor prognosis in colorectal cancer. *Medical Oncology*.

[116] Chen M. L., Liang L. S., Wang X. K. (2012). MiR-200c inhibits invasion and migration in human colon cancer cells SW480/620 by targeting ZEB1. *Clinical & Experimental Metastasis*.

[117] Alvarez-Díaz S., Valle N., Ferrer-Mayorga G. (2012). MicroRNA-22 is induced by vitamin D and contributes to its antiproliferative, antimigratory and gene regulatory effects in colon cancer cells. *Human Molecular Genetics*.

[118] Wang L.-G., Gu J. (2012). Serum microRNA-29a is a promising novel marker for early detection of colorectal liver metastasis. *Cancer Epidemiology*.

[119] Zhang Y., He X., Liu Y. (2012). MicroRNA-320a inhibits tumor invasion by targeting neuropilin 1 and is associated with liver metastasis in colorectal cancer. *Oncology Reports*.

[120] Okamoto K., Ishiguro T., Midorikawa Y. (2012). MiR-493 induction during carcinogenesis blocks metastatic settlement of colon cancer cells in liver. *The EMBO Journal*.

[121] Sakai H., Sato A., Aihara Y. (2014). MKK7 mediates miR-493-dependent suppression of liver metastasis of colon cancer cells. *Cancer Science*.

[122] Liu X., Zhang Z., Sun L. (2011). MicroRNA-499-5p promotes cellular invasion and tumor metastasis in colorectal cancer by targeting FOXO4 and PDCD4. *Carcinogenesis*.

[123] Cui Z., Tang J., Chen J., Wang Z. (2014). Hsa-miR-574-5p negatively regulates MACC-1 expression to suppress colorectal cancer liver metastasis. *Cancer Cell International*.

